# Structural characterization of the RH1-LZI tandem of JIP3/4 highlights RH1 domains as a cytoskeletal motor-binding motif

**DOI:** 10.1038/s41598-019-52537-3

**Published:** 2019-11-05

**Authors:** Fernando Vilela, Christophe Velours, Mélanie Chenon, Magali Aumont-Nicaise, Valérie Campanacci, Aurélien Thureau, Olena Pylypenko, Jessica Andreani, Paola Llinas, Julie Ménétrey

**Affiliations:** 1 Institute for Integrative Biology of the Cell (I2BC), CEA, CNRS, Univ. Paris-Sud, Université Paris-Saclay, 91198 Gif‐sur‐Yvette, cedex France; 2 Synchrotron SOLEIL, l’Orme des Merisiers, F-91410 Saint Aubin, France; 3 Structural Motility Team, Institut Curie, PSL Research University, CNRS, UMR 144, Paris, France

**Keywords:** Cytoskeletal proteins, Bioinformatics, SAXS

## Abstract

JIP3 and JIP4 (JNK-interacting proteins 3 and 4) are adaptors for cargo recruitment by dynein/dynactin and kinesin1 motors. Both are dimers that are stabilised by two sections of leucine zipper coiled coils. The N-terminal Leucine Zipper I (LZI) belongs to a section that binds dynein-DLIC and kinesin1-KHC, whilst the medial Leucine Zipper II (LZII) binds dynactin-p150glued and kinesin1-KLC. Structural data is available for the LZII, but the LZI section is still uncharacterized. Here we characterize the N-terminal part of JIP3/4 which consists of an RH1 (RILP homology 1) domain followed by the LZI coiled coil using bioinformatical, biophysical and structural approaches. The RH1-LZI tandem of JIP3 associates as a high affinity homodimer exhibiting elongated alpha-helical fold. 3D homology modelling of the RH1-LZI tandem reveals that the kinesin1-KHC binding site mainly overlaps with the RH1 domain. A sequence comparison search indicates that only one other protein family has RH1 domains similar to those of JIP3/4, the RILP (Rab-interacting lysosomal protein) family which consists of adaptor proteins linking Rab GTPases to cytoskeletal motors. RILPL2 is recruited through its RH1 domain by the myosin 5a motor. Here, we showed that the RH1 domain of JIP3 also interacts with myosin 5 A *in vitro*, highlighting JIP3/4 as possible myosin 5a adaptors. Finally, we propose that JIP3/4 and RILP family members define a unique RH1/RH2-architecture adaptor superfamily linking cytoskeletal motors and Rab GTPases.

## Introduction

C-Jun N-terminal kinase (JNK) interacting proteins 3 and 4 (JIP3 and JIP4; JIP3/4) were first identified as scaffolds for JNK and p38 Mitogen-Activated Protein Kinase (MAPK) signalling modules^1–3^. Subsequently, they were also found to be adaptors for two microtubule-based molecular motors which are involved in intracellular transport of various cargos, as organelles, vesicles, mRNA complexes or proteins^4^. The recruitment of JIP3/4 by kinesin1^5–7^ and dynein-dynactin complex^8,9^ allows their bidirectional transport, as well as that of their associated partners or motor cargos, in opposite directions along microtubules. Motor-driven transport of JIP3/4 and its associated partners is involved in many cellular processes, including axonal outgrowth, transport and damage signalling, muscle development, endosomal and mitochondrial transport, as well as in pathologies such as neurological diseases and cancer^5,8–16^. More recently, JIP3/4 were established as specific effectors for Arf/Rab GTPases which are master regulators of intracellular transport, connecting membranes to cytoskeleton motors or adaptors^9,17,18^. The interaction of JIP3/4 with Arf6 which is involved in the actin cytoskeleton and membrane trafficking organization^19^, regulates endosomal movement during cytokinesis^9^, fast endocytic recycling^20^ and endosomal tubule for MT1-MMP exocytosis in cancer invasion^16^, as well as macropinocytosis, an actin-driven form of clathrin-independent endocytosis^21^. The interaction of JIP3/4 with Rab36 which controls retrograde melanosome transport in melanocytes^22^, regulates neurite outgrowth^18^. Thus, JIP3/4 are versatile scaffolds that regulate numerous and various intracellular transport functions.

**Figure 1:**
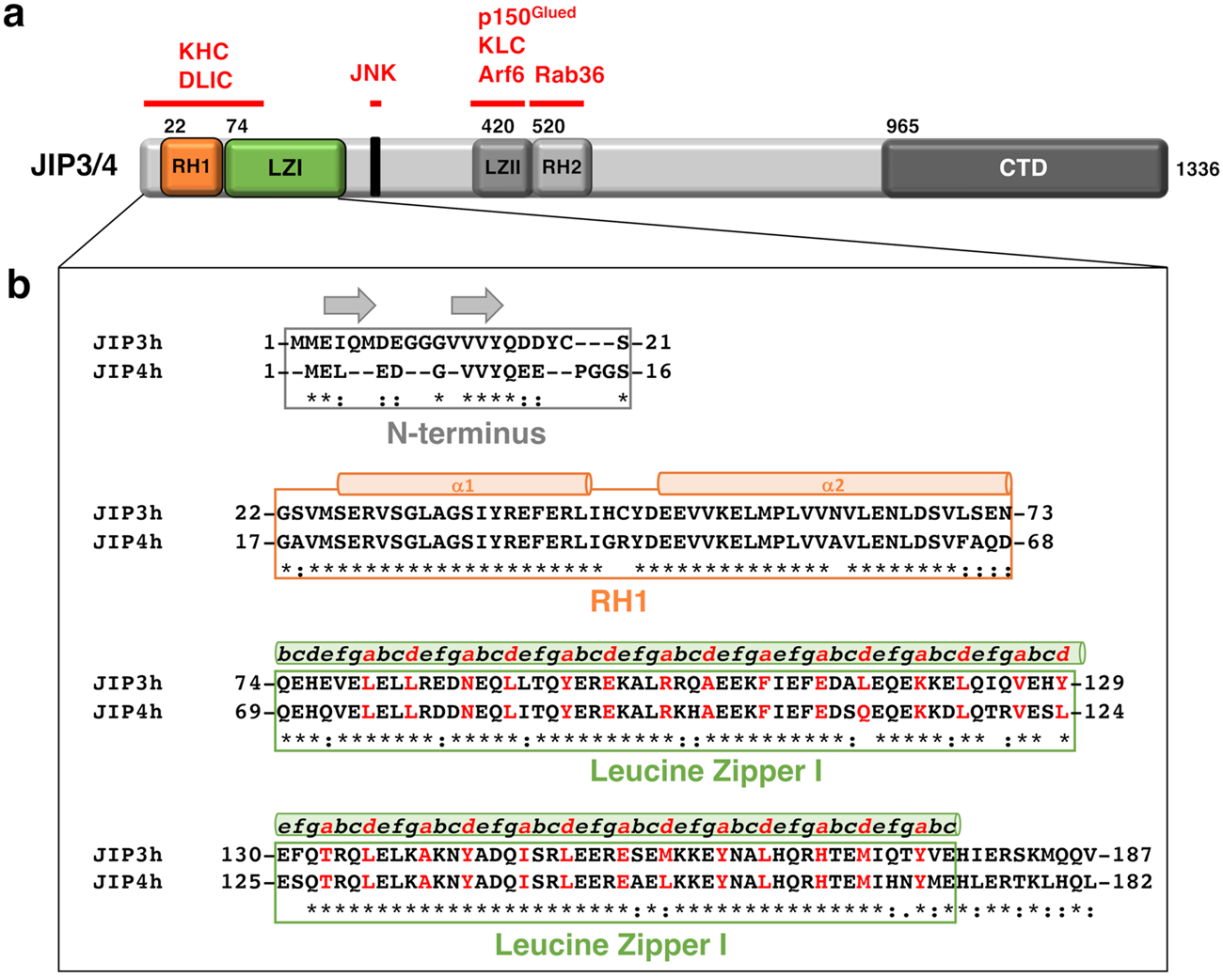
The organization of the RH1-LZI tandem of JIP3/4. (**a**) Schematic representation of the full JIP3/4 proteins. Sequence limits of JIP3 are indicated at the beginning of the known domains. RH1 and RH2: RILP homology 1 and 2; LZI and LZII: Leucine Zipper I and II; CTD: C-Terminal Domain. JNK: c-Jun N-terminal Kinase. The binding partners of JIP3/4 are indicated in red. **(b)** Sequence alignment of the N-terminal part consisting of the RH1-LZI tandem of human JIP3 and JIP4 homologs (NP_055948.2 and NP_001123999.1 accession numbers, respectively). Secondary structures are indicated above the sequence alignment. The repeated pattern ‘*abcdefg*’ of the heptad repeats of the Leucine Zipper I (LZI) is indicated above the sequence alignment with the characteristic hydrophobic residues at position ‘*a*’ and ‘*d*’ indicated in red.

JIP3/4 are large dimeric molecules encompassing several highly conserved regions among which are two coiled coils known as Leucine Zipper I and II (Fig. 1a). The Leucine Zipper II (LZII) coiled coil located in the median part of JIP3/4 is well characterized. It encompasses the binding sites for (i) the light chain of kinesin 1 (KLC)^6,9^, (ii) the p150^glued^ subunit of the dynactin complex^8,9^ and (iii) the small GTPase Arf6^9,23^. Crystal structures of JIP3/4-LZII bound to Arf6 and to KLC were determined^23,24^ and gave crucial insights to better understand how this protein-protein interaction module recognizes its partners. In contrast, less is known about the Leucine Zipper I (LZI) coiled coil and, more generally, about the N-terminal part of JIP3/4. Valeria Cavalli and colleagues established that the heavy chain of kinesin1 (KHC), directly and independently of its interaction with KLC, binds to the N-terminal part of JIP3^7^. Specifically, using GST-pulldown assays on mouse brain lysates, the region of JIP3 required to promote KHC interaction was narrowed down to a 30 residues-stretch composed of aa 50–80^7^. They determined that JIP3 activates KHC for transport and enhances its motility along microtubule^7^; such motility regulation is critical for axon elongation and regeneration^14^. In addition, the N-terminal part (aa. 1–240) of the *Caenorhabditis elegans* JIP3 protein, UNC-16, was identified as the binding site for the dynein light intermediate chain (DLIC) by yeast two-hybrid screen and confirmed by immunoprecipitation assays^25^. UNC-16/JIP3, associated with kinesin1 are required for localization of DLIC at the plus ends of nerve process microtubules and for retrograde transport of various axonal proteins in C. elegans^25^. To date, no structural characterization has been undertaken to describe the N-terminal part of JIP3/4 proteins which also represents also a protein-protein interaction module.

To characterize the N-terminal part of JIP3/4 which encompasses a RH1 (RILP homology 1) domain followed by the leucine zipper I (LZI) coiled coil, we perform bioinformatical, biophysical and structural characterizations of this region. A homology model structure of the RH1-LZI tandem of JIP3 was computed and assessed against experimental biophysical and SAXS data. Also, sequence comparison searches were performed to identify proteins exhibiting an RH1 domain similar to that of JIP3/4. Only RILP (Rab-interacting lysosomal protein) family members (RILP, RILPL1 and RILPL2), which are adaptor proteins linking Rab GTPases to cytoskeletal motors were identified. Interestingly, RILPL2 is recruited by myosin 5a through its RH1 domain, raising the question of the ability of the RH1 domain of JIP3/4 to interact with this actin-based motor. We investigate this question experimentally using two distinct binding assay approaches *in vitro*. Finally, we provide evidence that the RH1 domain is a cytoskeletal motor-binding motif and that both JIP3/4 and RILP family members define a unique RH1/RH2-architecture adaptor superfamily linking cytoskeletal motors and Rab GTPases.

## Results

### Sequence analysis of the N-terminal part of JIP3/4

Phylogenetic analysis of JIP3 and JIP4 homologs shows that JIP3/4-like homologs can be found in vertebrates (as well as insects and nematodes), while a single homolog of JIP3/4 (named JIP3/4-like) can be identified in invertebrates (Supplementary Fig. S1a)^26^. Sequence analysis of human JIP3 and JIP4 (NP_055948.2 and NP_001123999.1 accession numbers, respectively) indicates that these two homologs are close, sharing 56% sequence identity and several highly conserved regions (Fig. 1a). The N-terminal part consisting of residues [1–187] of JIP3 and [1–182] of JIP4 is one of these highly conserved regions with sequence identity reaching 75% (Fig. 1b and Supplementary Fig. S1b). Secondary structure predictions performed on the N-terminal part of both JIP3 and JIP4, using the GeneSilico Metaserver^27^, reveal an overall α-helical structure, except at the extreme N-terminus which is less structured and exhibits 2 and 1 small β-strands, respectively (Supplementary Fig. S2). The extreme N-terminus of JIP3 and JIP4 (residues [1–21] and [1–16], respectively) exhibits some conservation, despite differences in sequence length (Fig. 1b and Supplementary Fig. S1b). Beyond the extreme N-terminus, JIP3 and JIP4 exhibit a long coiled coil, namely the Leucine Zipper I (LZI). Using Paircoil software^28^, the coiled coil was delimited to residues [74–177] for JIP3 and residues [69–172] for JIP4 (consisting of 14 heptad repeats) sharing 82.3% sequence identity between the two homologs (Fig. 1b). Interestingly, a RH1 (RILP homology 1) domain was identified by the ScanProsite tool^29^ in both JIP3 and JIP4 proteins. The RH1 domain consists of residues [22–73] and [17–68], respectively in JIP3 and JIP4 sharing 84.6% sequence identity between the two homologs (Fig. 1b).

To search for remote homologs of the RH1 domain of the human JIP3 protein in the PDB^30^ and Pfam^31^ databases, as well as in the human proteome (Supplementary Table S1), we used the HHpred webserver^32,33^. In the PDB database, only mouse RILPL2 (PDB code 4KP3_D) was identified as a high confidence match (HHsearch probability > 95%). Despite the low sequence identity (around 20%), the high HHsearch probability (99.7%) as well as the consistent matching of secondary structures between the two proteins strongly support the structural homology. High confidence matches in the human proteome include only isoforms of the JIP3 and JIP4 proteins, as well as the RILP, RILPL1 (RILP-like 1) and RILPL2 (RILP-like 2) proteins: all display HHsearch probabilities >99%. Other matches are less confident (HHsearch probability <94%) and often align to shorter portions of the domain. Proteins matched for model species in InterPro family IPR019143 (corresponding to PF09744)^34^ also show the same homologs. An HHpred search starting from the sequence of the RH1 domain of RILPL2 (PDB code 4KP3_D) led to the identification of the same homologs. This reveals that RH1 domains are found only in JIP3/4 and in RILP family proteins.

Overall sequence analysis of the RH1 domain between JIP3/4 and RILP family proteins reveals that profile-profile alignment (HHsearch) identified strong homology relationships among these proteins (HHsearch probabilities >99%), while classical sequence-sequence alignment analysis showed low sequence identities. Indeed, RH1 domain sequence identities among RILP family proteins are low (35–40% identity) and even lower between JIP3/4 and RILP family proteins (20–35% identity). Interestingly, the RH1 domain, like the PH domain for instance^35^, share structural homology, whereas they exhibit low sequence homology. Such low sequence homology constitutes a determinant for versatility in partner recognition.

### Biophysical characterization of the N-terminal part of JIP3

**Figure 2:**
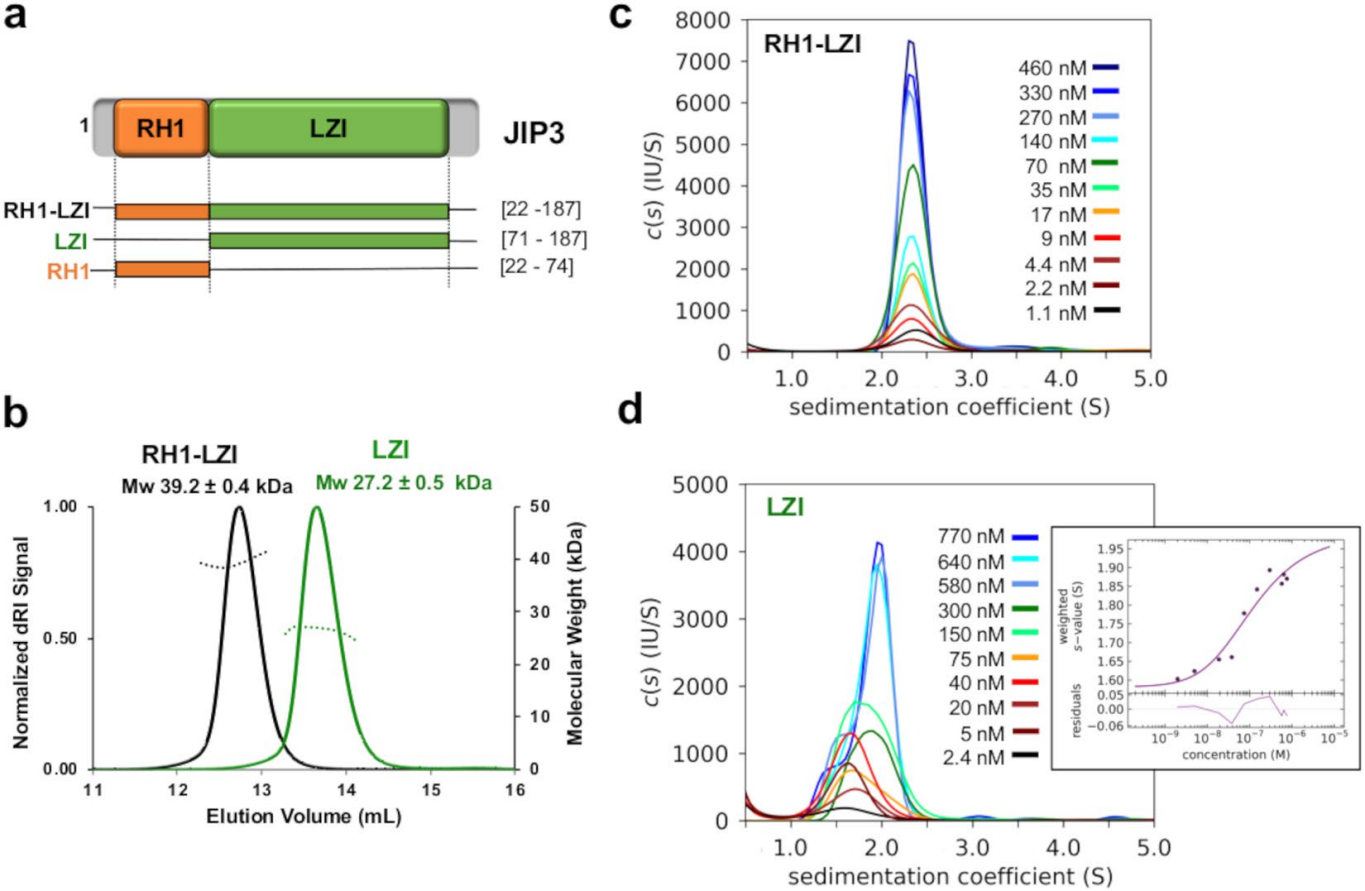
Biophysical characterization of the N-terminal part of JIP3. (**a)** Schematic representation of the different fragments of the N-terminal part of JIP3 used in this study. **(b)** Superposition of the MALS curves for RH1-LZI (black) and LZI (green) fragments of JIP3. **(c,d)** Characterization of the self-association of the JIP3-Nter dimers using fluorescence-detected sedimentation velocity (SV-FDS). Dilution series with fluorescent label for the **(c)** JIP3-RH1-LZI fragment and **(d)** JIP3-LZI fragment. Sedimentation coefficient distributions c(s) and for the LZI fragment the resulting sw isotherms (inset) from integration of c(s) are shown. In the isotherm plots, solid circles are the sw data from the dilution series, and the solid line is the best-fit isotherm.

In order to characterize the N-terminal part of JIP3 and JIP4, we conceived different fragments of both isoforms (Fig. 2a, Table 1 and Supplementary Fig. S3a and Table S2). The longest fragment consists of the RH1 domain followed by the LZI coiled coil without the extreme N-terminus (RH1-LZI fragment), while two shorter fragments consist of the LZI coiled coil alone (LZI fragment) or the RH1 domain alone (RH1 fragment). RH1-LZI and LZI fragments of both JIP3 and JIP4 were soluble (Supplementary Fig. S3b), while RH1 fragments were insoluble, revealing that in the absence of the LZI coiled coil, the RH1 domain is unstable.

**Table 1:** Biophysical characterization of the N-terminal part of JIP3.

Fragment			MALS			CD (%)				DSC
name	Limit	Mw* (kDa)	Mw (kDa)	Rh (nm)	f/f0	α-helix	β-sheet	Turn/Rand.	222/208 ratio	Tm (°C)
**JIP3**										
RH1-LZI	[22–187]	20.3	39.2 ± 0.4	4.1 ± 0.3	1.8	81.1	5.9	12.8	1.06	49.2
LZI	[71–187]	14.8	27.2 ± 0.5	3.5 ± 0.2	1.7	74.4	6.5	19.2	1.07	35.4
RH1	[22–74]	6.4	N.D.	N.D.	N.D.					

N.D. Not Determined; Rand., Random; *Mw is calculated considering the remaining sequence of the protease site after cleavage.

Different biophysical approaches were used to characterize, in solution, soluble JIP3/4 fragments. We determined the oligomeric state of JIP3 and JIP4 fragments, using size exclusion chromatography coupled with Multiple Angle-Light Scattering (SEC-MALS). For JIP3, RH1-LZI and LZI fragments eluted in single peaks that contain species with a molecular mass of 39.2 ± 0.4 and 27.2 ± 0.5 kDa, respectively (Table 1 and Fig. 2b), indicating a dimeric form in solution for both fragments. Also, QELS measurements determine a hydrodynamic radius (Rh) of 4.1 ± 0.3 and 3.5 ± 0.2 nm, for RH1-LZI and LZI fragments respectively (Table 1). These Rh values indicate that the RH1-LZI fragment is more elongated than the LZI. For JIP4, SEC-MALS experiments showed that both RH1-LZI and LZI fragments elute in single, but broad peaks that contain species with a molecular mass of 57.8 ± 0.3 and 41.2 ± 0.4 kDa, respectively (Supplementary Table S2 and Fig. S3c). Despite these Mw values, which correspond to almost 3 times the mass of the monomer, the shape of the peak and the fact that the equivalent fragments of JIP3 have a dimeric state, suggest that there is a fast-reversible equilibrium between a dimeric and a higher oligomeric state in solution for both fragments of JIP4. Due to this oligomeric state heterogeneity, we subsequently focused only on JIP3 fragments characterization. Circular Dichroism (CD) experiments showed that RH1-LZI and LZI fragments of JIP3 are mainly alpha-helical with 81.1% and 74.4% helix content, respectively (Table 1). These helix content percentages correspond to 135 and 87 residues, respectively for the RH1-LZI and the LZI fragments which are consistent with the consensus secondary structure prediction (132 and 94 residues, respectively; Supplementary Fig. S2). Further, the ratio of ellipticities between 222 and 208 nm for RH1-LZI and LZI fragments is greater than 1 (Table 1) which is indicative of the presence of interacting α-helices^36^. Differential Scanning Calorimetry (DSC) experiments gave a melting temperature (Tm) of 49.2 °C and 35.4 °C for RH1-LZI and LZI fragments of JIP3, respectively (Table 1). Furthermore, while RH1 domain alone is unstable, DSC data reveals that its presence stabilizes the LZI coiled coil since a 14 °C difference is observed between the RH1-LZI and the LZI fragments. Thus, RH1-LZI fragment of JIP3, as well as the LZI alone, are stable dimers and adopt all α-helical elongated shapes.

Sedimentation velocity (SV) analytical ultracentrifugation coupled with fluorescence detection (FDS) were used to determine the stability of the self-association of the LZI coiled coil in presence or absence of the RH1 domain. SV-FDS experiments confirmed that the JIP3-RH1-LZI fragment is a stable dimer in solution with a sedimentation coefficient of 2.3 S and exhibits an elongated shape with a f/f0 of 1.9 (Fig. 2c), consistent with the f/f0 calculated from SEC-MALS data (Table 1). Titration experiment then indicates that at low concentration (1.1 nM), the RH1-LZI fragment is still a stable dimer in solution with a sedimentation coefficient of 2.3 S (Fig. 2c). Therefore, we conclude that the Kd of the RH1-LZI dimer is sub-nanomolar. The JIP3-LZI fragment is also a dimer in solution with a sedimentation coefficient of 1.9 S and exhibits an elongated shape with a f/f0 of 1.8, consistent with the f/f0 calculated from SEC-MALS data (Fig. 2d and Table 1). However, titration experiment indicates that the sedimentation coefficient decreases and settles down to 1.7 S at the lowest concentrations. Similarly, the f/f0 decreases to 1.4 which no longer corresponds to an elongated shape. The Kd of the JIP3-LZI dimer is determined to 110 nM with a confidence interval from 65 nM to 185 nM (Fig. 2d). This data therefore indicates that the RH1-LZI fragment forms a more stable dimer than LZI does which is consistent with the Tm variation observed between these two fragments using DSC (Table 1 and Fig. 2d). Altogether, these biophysical characterizations reveal that the RH1 domain and the LZI coiled coil form a structural tandem since (i) in absence of the LZI coiled coil, the RH1 domain is unstable and (ii) in absence of the RH1 domain, the LZI coiled coil is less stable.

### Homology modelling of the N-terminal part of JIP3

**Figure 3:**
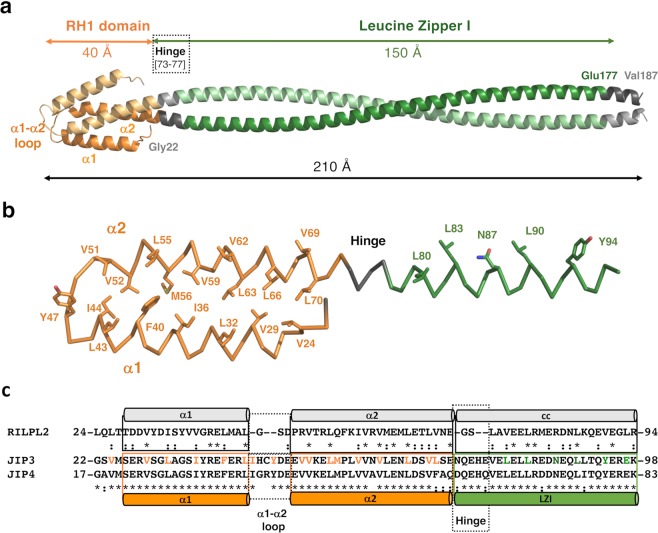
Homology modelling of the RH1-LZI tandem of JIP3. (**a)** Homology model of the RH1-LZI tandem of human JIP3. **(b)** One protomer of the JIP3-RH1-LZI model is shown in ribbon with hydrophobic residues indicated in sticks and labeled. The protomer is shown in the same orientation as in (**a**). Only the first three heptad repeats of LZI are shown for clarity. **(c)** Structure-based sequence alignment of RILPL2, JIP3 and JIP4. Secondary structures of both the crystal structure of RILPL2-RH1 and the homology model of JIP3-RH1 are indicated. Hydrophobic residues at the interface of the RH1 dimer are shown in orange, and those of the LZI dimer are shown in green on the JIP3 sequence.

Next, we sought to obtain 3D information on the N-terminal part of JIP3/4. Because our crystallization attempts have been unsuccessful so far, we computed a model of the RH1-LZI fragment of human JIP3. On the one hand, the RH1 domain (residues [22–73]) of JIP3 was modelled based on the crystal structure of the RH1 domain of RILPL2 (PDB code 4KP3^37^;), giving a very high confidence score (99.8%) using the Phyre2 web server^38^. The RH1 domain is a homodimer mediated by a four-helix bundle constituted by two anti-parallel helices from each protomer, with a length of 40 Å (Fig. 3a; helices α1 and α2 consist of residues [26–43] and [50–73], respectively). Hydrophobic residues are found at the dimer interface: (i) Val24 from the linker between the extreme N-terminus and the α1 helix, (ii) Val29, Leu32, Ile36, Phe40, Leu43, Ile44, Tyr47 from α1 helix and (iii) Val51, Val52, Leu55, Met56, Val59, Val62, Leu63, Leu66, Val69 and Leu70 from α2 helix (Fig. 3b). Sequence comparison between our model of the RH1 domain of JIP3 and the crystal structure of that of RILPL2 shows that most hydrophobic residues at the interface of the dimer are conserved (Fig. 3c). Due to sequence differences between JIP3 and RILPL2, the modelling of the α1-α2 loop is unclear. In RILPL2, the loop is short consisting of 3 residues, while in JIP3/4 this segment is longer with 3 extra residues (Fig. 3c). Here, we modelled the α1-α2 loop of JIP3 with 6 residues, but we cannot exclude that some of them contribute to extend α1 and/or α2 helices, as suggested by secondary structure predictions in which differences are observed at this position among the various prediction methods (Supplementary Fig. S2). In addition, at the beginning of the α2 helix of JIP3/4, a proline residue (well known as helix “breakers”) is found (Pro57 in JIP3), whereas it is absent in RILPL2. Thus, this proline residue might either induce a kink into the α2 helix or shorten it. To resolve these two issues, the experimental 3D structure of the RH1 domain of JIP3/4 will be required. On the other hand, the LZI coiled coil (residues [74–177]) of JIP3 was modelled as a parallel dimeric coiled coil using CCbuilder 2.0^39^. The LZI consists of a straight coiled coil of 150 Å-length (Fig. 3a) since no glycine or proline residues are observed in the sequence (Fig. 1b). It should be noted that while coiled coil prediction indicates the end of the LZI at residue 177, secondary structure predictions indicate that residues beyond 177 also adopt α-helical structure (Supplementary Fig. S2). Therefore, residues 178 to 185 were modelled as a continuity of the coiled coil, but we cannot exclude that these residues exhibit a different α-helical structure. Finally, to model the RH1 domain relative to the LZI coiled coil, we took advantage of the known 3D structure of the RH1-cc tandem of RILPL2 (PDB code 4KP3^37^). The sequences corresponding to the hinge between the RH1 domain and the LZI coiled coil is longer than that found in RILPL2 (Fig. 3c) and exhibits no glycine/proline residues. Secondary structure predictions of both JIP3 and JIP4 indicate that these residues adopt an α-helical structure (Supplementary Fig. S2). Thus, we anticipate that the hinge between the RH1 domain and the LZI coiled coil is structured as a α-helix and consequently that the RH1 domain is orientated straight in relation to the main axis of the LZI coiled coil. This model of the RH1-LZI tandem of JIP3 reaches a total length of 210 Å for a thickness of 20 Å (Fig. 3a). The predicted Rh and Cs of this model, calculated by the Hydropro program^40^, are 4.4 nm and 2.2 S, respectively, which are like those measured using SEC-MALS (Table 1) and SV-FDS (Fig. 2c). Thus, our structural model of the RH1-LZI tandem is concordant with our biophysical data.

### Conformational properties of the RH1-LZI tandem of JIP3 from SAXS studies

**Figure 4:**
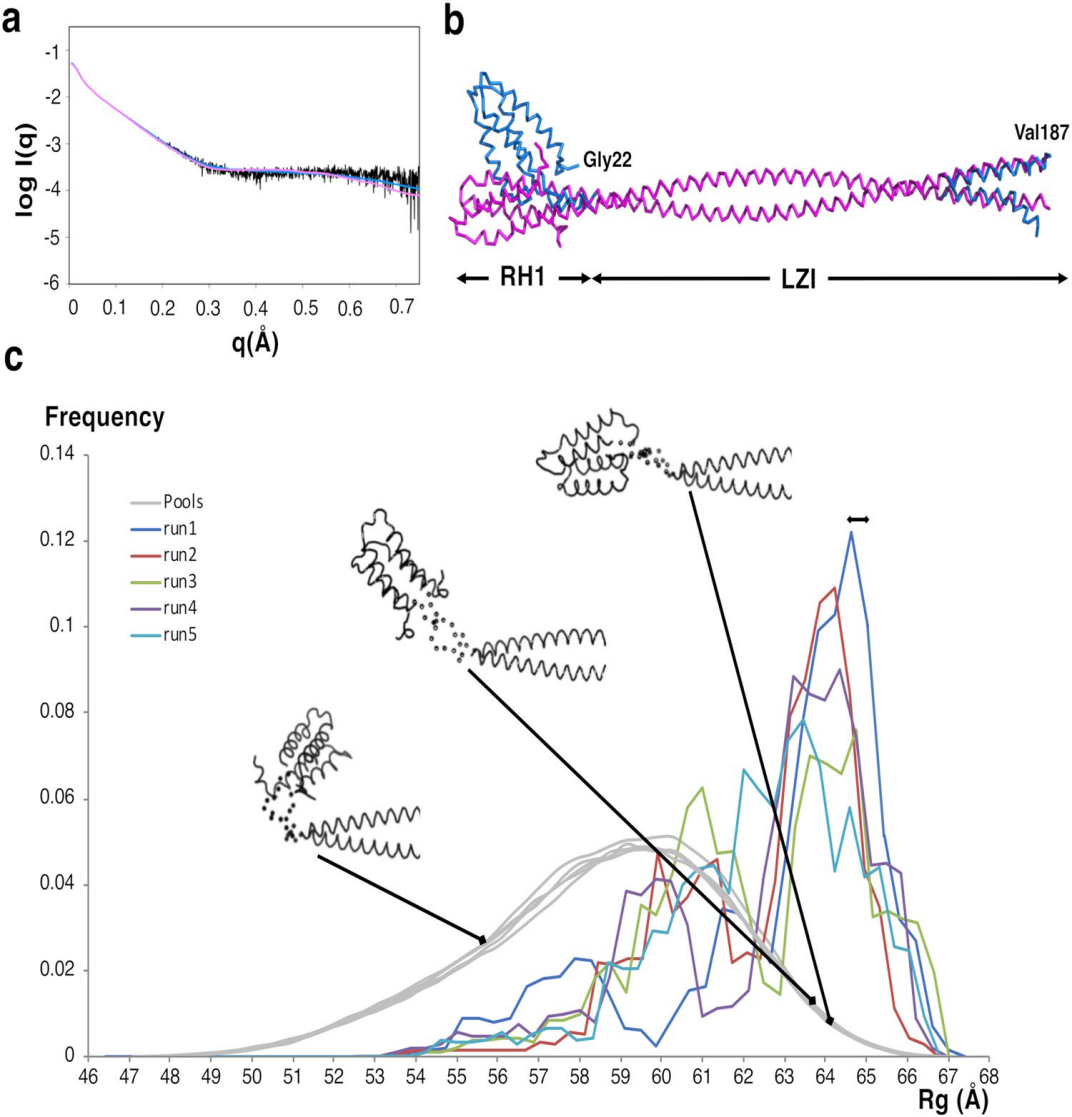
SAXS studies of the RH1-LZ1 tandem of JIP3. (**a**) Comparison of the experimental SAXS profile (black) and the theoretical profiles calculated by CRYSOL of the initial model (pink) and the best DADIMODO refined model (blue). **(b)** Superposition of the initial model (pink) and the best refined model (χ^2^ = 1.271; blue) from five independent runs of DADIMODO. **(c)** Radius of gyration (Rg) distributions derived from the ensemble optimization method (EOM) analysis on 5 independent runs. Three models with different Rgs are displayed. The black bar on top represents the Rg values of Dadimodo models (64.6 to 65 Å).

To further assess the model of the N-terminal part of JIP3, we collected small angle X-ray scattering **(**SAXS) data on the RH1-LZI fragment, as well as on the LZI fragment alone for comparison (Supplementary Fig. S4a). For the RH1-LZI fragment, the Guinier fit from q~0.0085 to 0.018 gives a Rg value of 63.4 ± 2.9 Å without indication of aggregation (Supplementary Fig. S4b and Table S3). The molecular mass estimated from SAXS data with the program SAXSMoW^41^ is 42.3 kDa, which corresponds to a dimeric state of the RH1-LZI fragment of JIP3, as previously determined using SEC-MALS (Supplementary Table S3). The dimensionless Krakty plot^42^ shows a bell-shaped curve indicating that the RH1-LZI fragment is a folded molecule, with a maximum of 4.1 at 7.4 qRg (Supplementary Fig. S4c). For the LZI fragment, the maximum is 3.4 at 6.8 qRg (Supplementary Fig. S4c) indicating that the LZ1 fragment is shorter than the RH1-LZI fragment. For comparison, the maximum of a fully folded globular protein with one domain is close to 1.1 at 1.75 qRg. Thus, the shift from these values for the LZI and the RH1-LZI fragments of JIP3 is an indication of an elongated shape. Interestingly, the course of the dimensionless Krakty plots at high q indicates some flexibility of the RH1-LZI fragment (Supplementary Fig. S4c). The pair distribution function P(r) exhibits a typical shape of a long rod with a maximum around 17 Å and a Dmax of 231 Å (Supplementary Fig. S4d), which is concordant with the thickness and the elongated shape of our model. For comparison, the pair distribution function P(r) of the LZI alone also exhibits a shape typical of a long rod with a maximum around 17 Å, but a Dmax of 198 Å (Supplementary Fig. S4d). Lastly, the Rg determined from the distance distribution function is 66.1 ± 0.4 Å for the RH1-LZI fragment which is close to those calculated from the Guinier fit (Supplementary Table S3). For comparison, the predicted Rg for the RH1-LZI model of JIP3, calculated by the Hydropro program^40^ is 64 Å which is quite similar to the experimental Rg determined using SAXS. Thus, SAXS analysis of the RH1-LZI fragment is concordant with our previous biophysical characterization. The model of the RH1-LZ1 tandem of JIP3 fits the SAXS data with a χ^2^ of 2.108 (Supplementary Fig. S4c). To further refine the model against the experimental data, we used the DADIMODO program which allows for the release of structural restraints at specific defined regions^43,44^. We released structural restraints at regions that we expected to exhibit some flexibility: (i) the N-terminus of the RH1 domain, (ii) the C-terminus of the LZI coiled coil and (iii) the hinge between the RH1 domain and the LZI coiled coil. Five independent refined models provide an improved fit against SAXS data with χ^2^ ranging from 1.271 to 1.312 (Fig. 4a). The most important characteristic of the refined models is a movement of the RH1 domain relative to the LZI coiled coil indicating some flexibility at the hinge between the RH1 domain and the LZI coiled coil (Fig. 4b). In order to estimate this flexibility degree, the Rg distribution variability of the JIP3-RH1-LZ1 model was calculated using EOM 2.1^45^. The Rg distribution of 5 independent runs of EOM indicates a tendency to extended conformation (run 1 to 5, Rg = 64.4 Å) in solution compared to aleatory conformations (pools) (Fig. 4c), which agrees with the DADIMODO models (Rg = 64.6 to 65 Å). Thus, overall SAXS data indicates that the RH1-LZI tandem is mostly in an extended conformation in solution, and the hinge between the RH1 domain and the LZI coiled coil could be bent to some degree.

### KHC-binding site overlaps with the RH1 domain of JIP3/4

**Figure 5:**
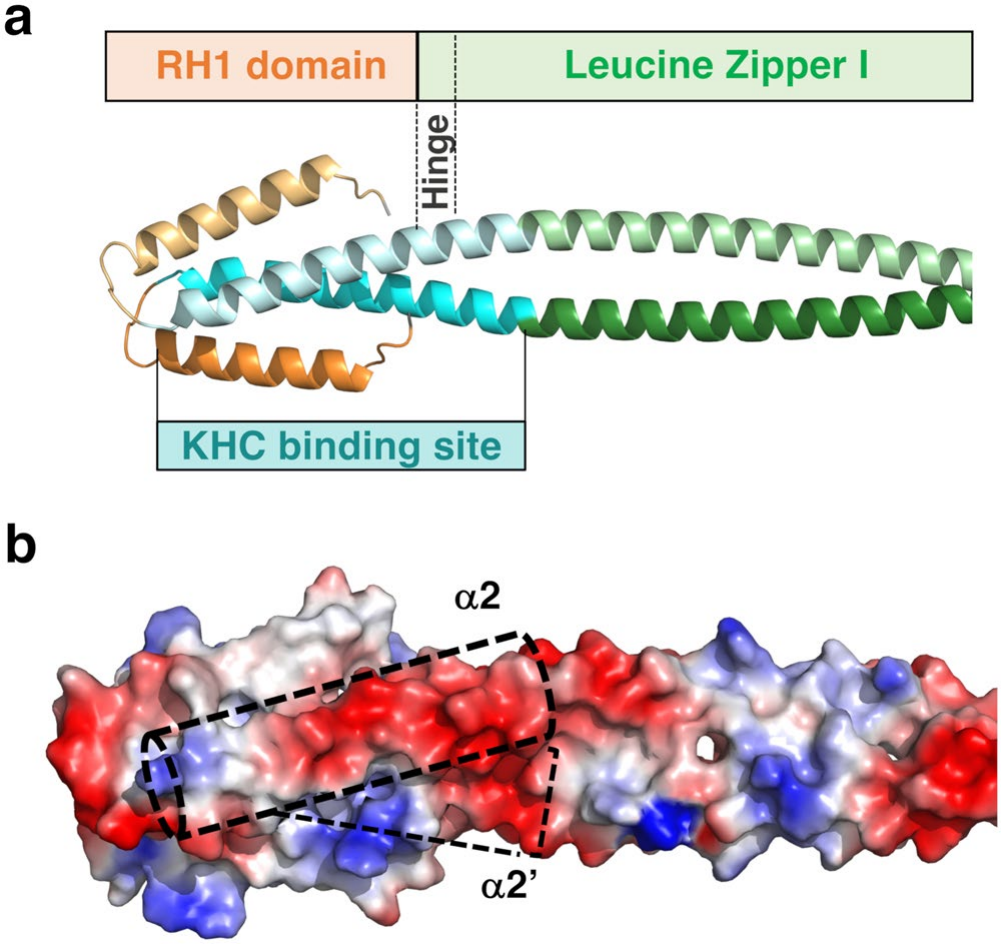
KHC-binding site overlaps with the RH1 domain of JIP3/4. (**a)** Homology model of JIP3-RH1-LZI tandem is shown in cartoon with the same color code as in Fig. 1 and the KHC-binding region (aa. 50–80) indicated in cyan. Only the first six heptad repeats of LZI are shown for clarity. **(b)** A surface representation of the JIP3-RH1-LZI model is shown in the same view as in (a). Acidic and basic residues are colored in red and blue, respectively; other residues are colored in white. The position of KHC-binding sites (aa 50–80) are indicated in dashed cyan boxes.

The region of JIP3 required for KHC interaction has been previously narrowed down to a 30 residues-stretch composed of residues [50–80]^7^. Interestingly, the JIP3-RH1-LZI model that we computed, based on bioinformatics analysis and validated by biophysical and structural data, shows that this region corresponds to the complete α2 helix (residues [50–72]) of the RH1 domain, as well as a few residues beyond consisting of the first heptad repeat (residues [73–80]) of the LZ1 coiled coil that encompasses the hinge region (Fig. 5a). Among these 30 residues, nine are acidic residues (Fig. 3c) which form a negative surface (Fig. 5b) that should suitably complement positive electrostatic potential (pI > 10) of the tail of KHC. Interestingly, three acidic residues are found beyond residue 80 in the LZI coiled coil, Glu81, Glu85 and Glu88 (Fig. 3c) which extend the negatively charged surface (Fig. 5b). Similarly, acidic residues are found before residue 50 in the α1-α2-loop, Asp48 and Glu49 (Fig. 3c) which also form a negative surface (Fig. 5b) that could be targeted by KHC-tail. Finally, we cannot exclude that residues from the α1 helix (residues [26–43]) of the RH1 domain, though not critical^7^, might also be involved in the interaction, since they are spatially close (Fig. 5). Overall, this structural analysis reveals that the KHC-binding site mainly overlaps with the RH1 domain of JIP3/4.

### Does myo5a-GTD interact with the RH1 domain of JIP3/4?

**Figure 6:**
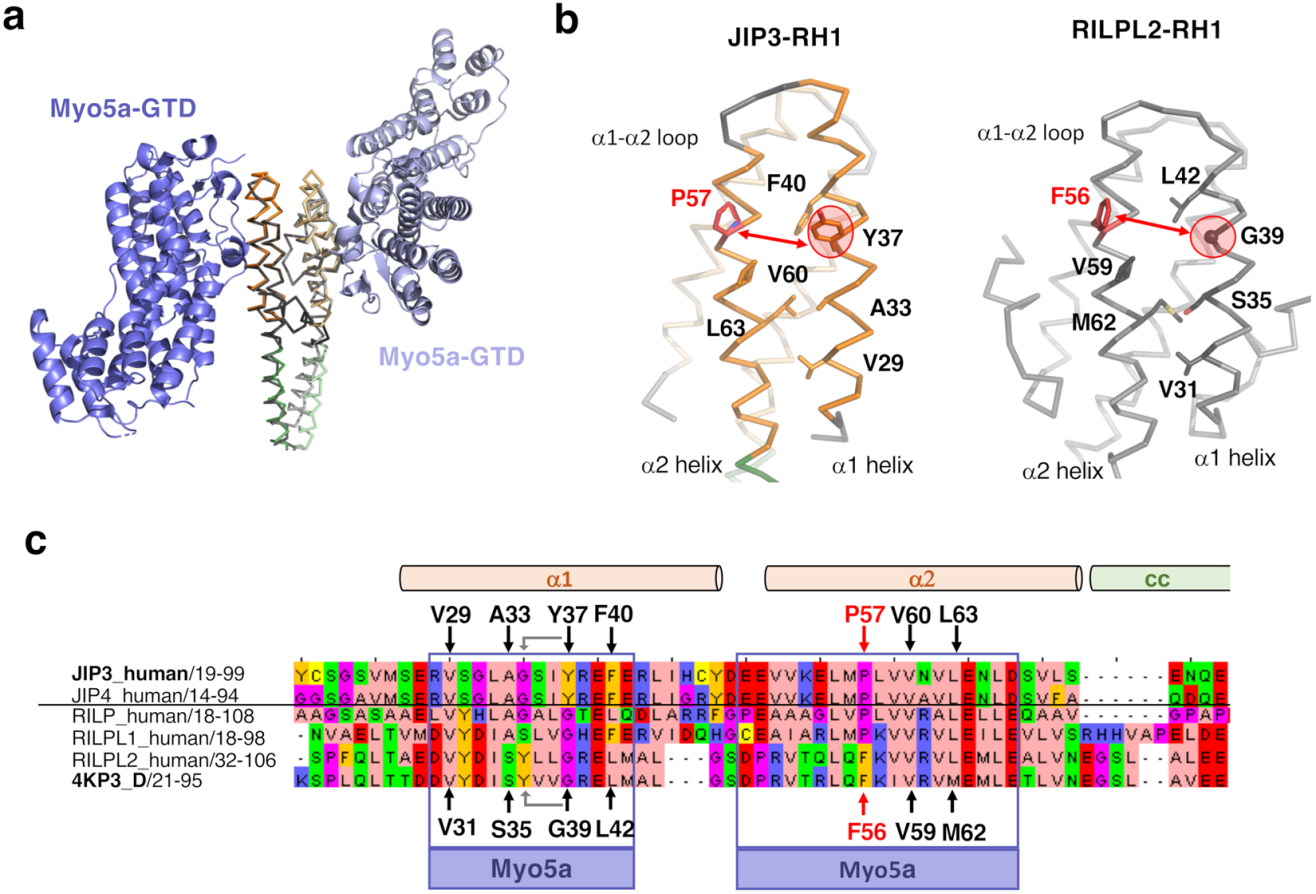
Structural analysis of the interaction between the RH1 domain of JIP3 and myo5a-GTD. (**a)** Superposition of the JIP3-RH1-LZI homology model (orange and green) on the crystal structure of the RH1 domain of RILPL2 (dark grey) bound to myo5a-GTD (shown in cartoon in blue) (PDB code 4KP3). **(b)** Myo5a-GTD-binding interface on JIP3-RH1domain (left, in orange) and RILPL2-RH1 domain (right, in grey). Residues in interaction with myo5a-GTD are shown in sticks and labeled. **(c)** Alignment of the RH1 domain between human JIP3 and JIP4 sequences, human RILP, RILPL1 and RILPL2 sequences and the mouse RILPL2 sequence as present in PDB 4KP3_D. This alignment was derived from HHpred results. Regions involved in the interface with myo5a-GTD in 4KP3 are indicated by purple boxes. In red is indicated a possible complementary sequence difference between RILPL2 and JIP3.

RILPL2 which shares a RH1 domain-coiled coil (RH1-cc) tandem with JIP3/4, is recruited through its RH1 domain by the myosin 5a (myo5a) actin-based motor^46^. Therefore, we wondered if myo5a also interacts with JIP3/4 through its RH1 domain. The crystal structure of the RH1-cc tandem of RILPL2 bound to the globular tail domain (GTD) of myo5a (PDB code 4KP3^37^) shows that myo5a-GTD makes interactions with both α1 and α2 helices from one protomer of the RH1 domain of RILPL2; the coiled coil (cc) of the tandem is not involved in the interaction (Fig. 6a). The interface can be described by two sets of interactions: (i) a hydrophobic patch of RILPL2-RH1, mainly constituted by residues Val31, Ser35, Gly39, Leu42 from α1 helix and Val59 and Met62 from α2 helix (Fig. 6b,c, black arrows) mediates interactions with Val1498 and Leu1502 from myo5a-GTD and (ii) the Phe56 from the α2 helix of RILPL2-RH1 (Fig. 6b,c, red arrow) inserts its bulky side chain into a hydrophobic pocket formed at the myo5a-GTD surface^37^. Structure superposition of the RH1 domain of JIP3 with that of RILPL2 reveals that the first set of interactions might be conserved between JIP3-RH1 and myo5a-GTD (Fig. 6b,c). Val31 and Val59 residues of RILPL2 are conserved in JIP3 (Val29 and Val60, respectively), Ser35, Leu42 and Met62 are similar (Ala33, Phe40 and Leu63, respectively in JIP3), but Gly39 is different (Tyr37 in JIP3). However, a reverse substitution (Tyr36 in RILPL2 to Gly34 in JIP3) is found one helix turn before which might complement this sequence difference. Concerning the second set of interactions, the critical Phe56 in RILPL2 is replaced by a proline residue in JIP3/4-RH1 (Fig. 6b,c). Interestingly, this position is also a proline residue in RILPL1 and RILP proteins (Fig. 6c). Wei and al. showed that substitution of the RILPL2-Phe56 to a proline residue disrupts the interaction with myo5a-GTD, while substitution of RILP-Pro55 in phenylalanine renders the RILP-RH1 mutant capable of Myo5-GTD binding, even with a higher binding affinity than the wild-type RILPL2-RH1^37^. Consequently, since this position is occupied by a proline residue, we assume that the JIP3/4-RH1 domain cannot interact with myo5a-GTD. However, due to the low sequence homology (23% sequence identity) between RH1 domains of RILPL2 and JIP3, we cannot exclude that complementary sequence substitutions may nevertheless allow myo5a-GTD interaction.

**Figure 7:**
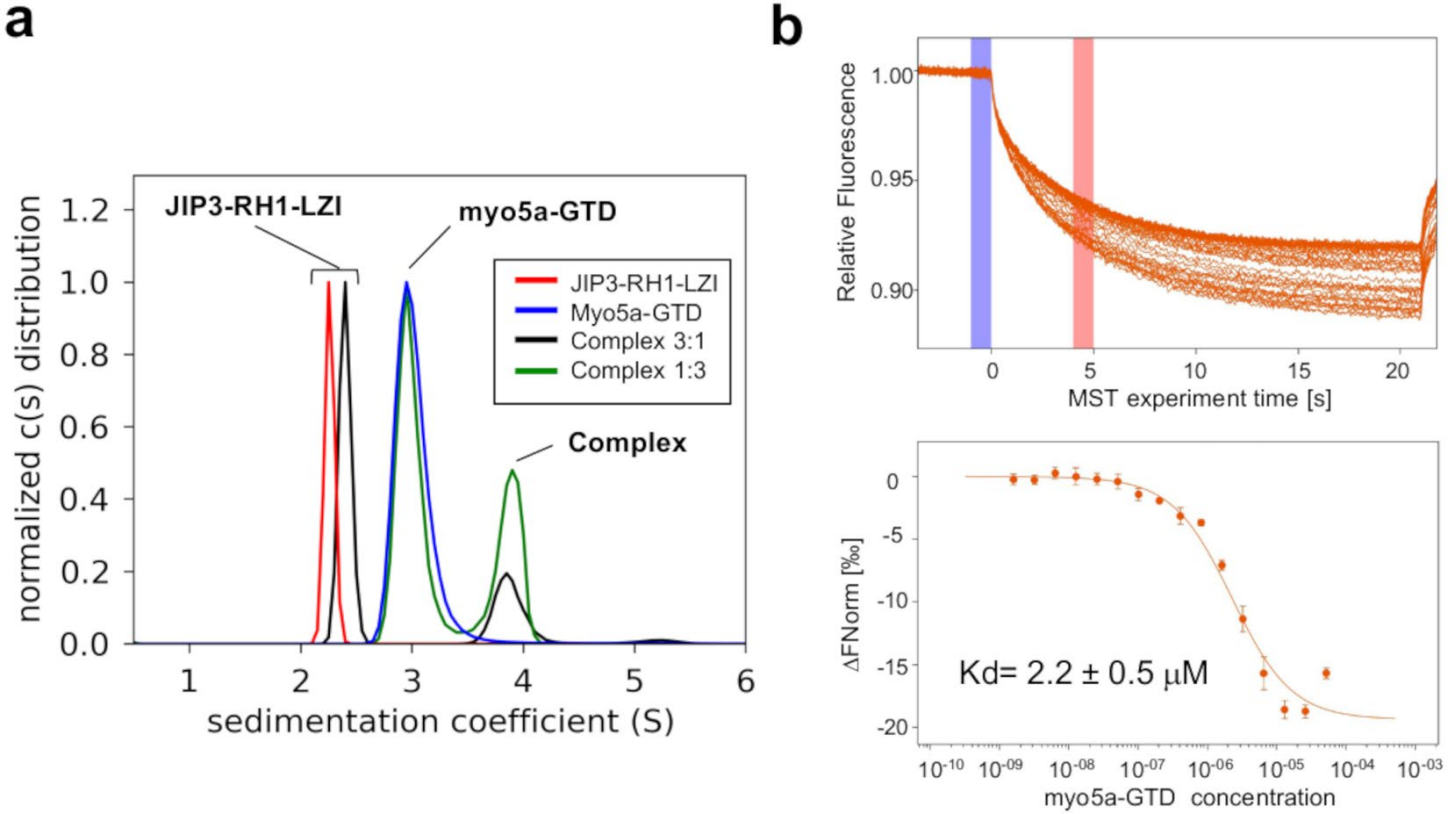
Formation of a complex between JIP3-N-ter and myo5a-GTD studied by AUC and MST (**a)** Complex formation of JIP3-RH1-LZI with myo5a-GTD studied by sedimentation velocity analytical ultracentrifugation. Continuous sedimentation coefficient distribution analysis of JIP3-RH1-LZI alone (red line), myo5a-GTD alone (blue line) and JIP3-RH1-LZI:myo5a-GTD mixtures (mixture 3:1, black line and mixture 1:3, green line). The total integrated signal of each c(S) distribution was normalized. **(b)** (Up) MST Traces, showing the ensemble of the 3 independent titration measurements. Vertical purple and pink bars show the initial and final analysis MST time. (Down) MST binding profile, where the ΔFnorm values are represented as a function of myo5A-GTD concentration. MST experiments were performed with labelled JIP3-RH1-lz3 (in orange) fragment and each titration sample is represented by a bullet and its variation from the merge of 3 independent replicates.

To our knowledge, no data has been reported on a direct interaction between JIP3/4 and myo5a, but both proteins are found on the same vesicles^47^ making their interaction possible in a cellular context. To experimentally assess the ability of the RH1 domain of JIP3 to interact with myo5a-GTD, we performed *in vitro* binding experiments. Sedimentation velocity analytical ultracentrifugation (AUC-SV) experiments indicated that a mixture of myo5a-GTD over an excess of JIP3-RH1-LZI shows two species: one with a sedimentation coefficient (Cs) of 2.4 S corresponding to the excess of JIP3-RH1-LZI and a second one with a higher Cs of 3.9 S (Fig. 7a), while a reverse mixture also shows two species: one with a Cs of 3.0 S corresponding to the excess of myo5a-GTD and a second one with the same higher Cs at 3.9 S (Fig. 7a). We conclude that the species with a Cs = 3.9 S corresponds to a myo5a-JIP3 complex. To further characterize this interaction, microscale thermophoresis (MST) experiments were performed to confirm the interaction and further delineate the binding interface. In order to compare with the binding affinity measured between RILPL2-RH1-cc and myo5a-GTD^37^, we generated a shorter fragment of JIP3 which is equivalent to that of RILPL2. The RH1-lz3 fragment of JIP3 consists of the RH1 domain followed by the three first heptad repeats of the LZI coiled coil (residues [22–100], Supplementary Fig. S5a). SEC-MALS experiment showed that this shorter fragment is dimeric (Supplementary Fig. S5b) revealing that truncation of the rest of the LZI coiled coils did not affect the overall structure of the fragment. MST data showed that myo5a-GTD binds to the labelled JIP3-RH1-lz3 with an affinity of 2.2 ± 0.5 µM (Fig. 7b) which is weaker than that of RILPL2-RH1-cc (Kd = 0.3 ± 0.05 µM, determined using ITC^37^;). Although there is often a difference in Kd between different biophysical approaches, a 7-fold difference indicates a significant variation in binding affinity. Thus, our binding data reveals that myo5a-GTD binds to the RH1-lz3 region of JIP3, *in vitro*. However, differences in binding affinity and sequence, such as the presence of a proline residue in JIP3/4 instead of the critical phenylalanine residue found in RILPL2^37^, suggest that the mode of recognition varies in detail between RILPL2 and JIP3/4. Further experiments are now needed to confirm that JIP3/4 is a binding partner of myo5a in a cellular context.

## Discussion

Our study reveals that the N-terminal part of JIP3/4 is not only the binding site for the kinesin1-KHC^7^ and Dynein-DLIC^25^ which are microtubule-based motors with opposite directionality, but also for myosin 5a, an actin-based motor. This highlights that the RH1-LZI tandem, like the LZII coiled coil located in the median part of the protein, are important and versatile protein-protein interaction modules for multiple cytoskeletal motors. Thus, JIP3/4 interact with kinesin1-KHC and kinesin1-KLC, independently at both the RH1-LZI and LZII binding sites, respectively^6,7,9^ and with dynein/dynactin complex (*via* DLIC and p150^glued^) also, respectively at both binding sites^8,9,25^, while JIP3 interacts with myosin 5a at the RH1-LZI binding site *in vitro* (this study). To our knowledge, no adaptor has yet been described as interacting directly with both microtubule- and actin-based motors. Interestingly, kinesin1-KLC and dynactin-p150^glued^ compete for their interaction on the LZII binding site of JIP3, as well kinesin1-KHC and myosin 5a both interact with the RH1 domain and one to three first heptads of the coiled coil that might be the basis for further competition. Such motor competition for JIP3/4 interaction, and thus JIP3/4 recruitment are potentially determinants for cargo transport regulation. Furthermore, JIP3/4 are not simply structural adaptors required for site of attachment, but also allosteric regulators for motor activation, like in the case of kinesin1-KHC^7^. Altogether, these studies show that JIP3/4 are critical regulatory “hubs” allowing the coordination of cargo transport between the microtubule and actin cytoskeletons.

In recent years, numerous structural data from X-ray crystallography or CryoEM reported cytoskeletal motors, such as kinesin1, dynein/dynactin and myosin5 motors^48–50^ bound to different type of adaptors^48–50^. Two distinct modes of adaptor recognition shared by multiple cytoskeletal motors have emerged and were recently summarized^48^. Unstructured peptide motifs (10 to 25 residues) represent the first structural motor-binding motif, like the globular tail binding motifs (GTBM) found in various myosin5 adaptors (for example Melanophilin (MLPH), Spir-1/2 or Granuphilin) or W-acidic and Y-acidic motifs found in various kinesin1-KLC adaptors (for instance in SKIP and JIP1, respectively). Dimeric coiled coils represent the second structural motor-binding motif, like the LZII coiled coil of JIP3/4 that is recognized by kinesin1-KLC or coiled coils found in various dynein/dynactin adaptors (for instance in HOOK3, BICD2 and JIP3). Here, we highlight a third structural motor-binding motif which is also recognized by multiple cytoskeletal motors, namely the RH1 domain. Several studies reported such motor-adaptor modes of recognition: (1) the RH1 domain of JIP3 was identified as the binding site for the heavy chain of kinesin1 (KHC) (this study^7^); (2) the RH1 domain of RILPL2 is the binding site of the GTD domain of myosin 5a^37,46^; (3) the 50 first residues of RILP, which encompasses its RH1 domain were proposed as critical for the interaction with the p150^glued^ subunit of dynactin complex^51–53^; and (4) the RH1 domain of JIP3 can interact *in vitro* with the GTD domain of myosin 5a (this study). In addition, dynein-DLIC interacts with both the N-terminal half of RILP encompassing the RH1-cc tandem^54,55^ and the N-terminal part (aa. 1–240) of UNC-16/JIP3 encompassing the RH1-LZI tandem^25^. However, further experiments are required to determine if the RH1 domain of both RILP and UNC-16/JIP3 are critical for these interactions. Altogether, this identifies the RH1 domain as a versatile structural cytoskeletal motor-binding motif.

**Figure 8:**
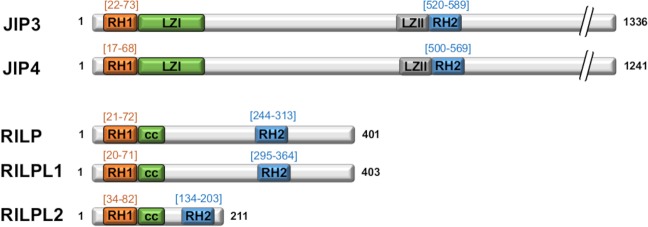
Architecture of proteins containing RH1-RH2 domains. RH1 and RH2 domain delimitation are indicated according to PROSITE domain definition for human sequences.

Finally, remote homology searches revealed that RH1 domains are found only in JIP3/4 and RILP families, defining one common characteristic of these motor adaptors. Interestingly, a second characteristic of these RH1 domain-containing proteins is the presence of a RH2 domain (RILP homology 2) (Fig. 8). The RH2 domains of JIP3 and RILPs were shown to recruit various Rab small GTPases^22,56^ which are critical regulators of intracellular transport connecting membrane vesicle to cytoskeleton motors or adaptors^17^. Thus, JIP3/4 and RILP family members constitute a unique RH1/RH2-architecture adaptor superfamily that links various Rab GTPases to multiple cytoskeletal motors allowing specific membrane association to directional movement.

## Materials and Methods

### Sequence analysis

To perform primary sequence analysis, human JIP3 and JIP4, with accession numbers NP_055948.2 and NP_001123999.1 respectively, were used. Sequence alignment of human JIP3 and JIP4 was performed using ClustalW^57^. Coiled coil primary sequence characteristics were analysed using paircoil2^28^. The phylogenetic study was performed using full-length sequences of JIP3 and JIP4 homologs retrieved using 2 iterations of InterEvolAlign^58^: one iteration against the OMA 2011 ‘Entire genomes’ database^59^, then another one against the RefSeq database^60^. Those full-length sequences were re-aligned using the MAFFT E-INS-i algorithm^61^. A phylogenetic tree was calculated using the SeaView graphical user interface^62^. First, a site selection was performed using Gblocks^63^ with options for less stringent selection. Then, the tree was computed with the PhyML program (version 3.1)^64^ using the LG substitution model (with optimized across-site rate variation using 4 substitution rate categories), empirical amino acid equilibrium frequencies and an optimized fraction of invariable sites. The final tree was visualized with Dendroscope 3^65^. The associated multiple sequence alignment was visualized with Jalview^66^.

The HHpred webserver^32,33^ with 1 iteration of HHblits against the uniclust30_2018_08 for query profile generation and all other parameters set to their default values was used to search for homologs of the sequence of the JIP3_MOUSE N-terminal region (positions 1–98) in the PDB database (PDB_mmcif70_10_Apr), the Pfam database (Pfam-A_v32.0) and the human proteome (Homo_sapiens_04_Jul_2017). A search with the same parameters was also performed using the sequence of PDB 4KP3_D as a query, for comparison purposes.

### Gene constructs, protein expression and purification

cDNAs encoding RH1-LZI (residues 22–187), LZI (residues 71–187) and RH1 (residues 22–74) fragments, respectively of mouse JIP3 (NP_038959.2), as well as RH1-LZI (residues 17–182, LZI (residues 67–182) and RH1 (residues 17–69) fragments, respectively of human JIP4 (NP_001123999.1) were cloned into the pnEAtHX^67^ (kind gift of Didier Busso, CEA, Saclay, France) plasmid between *NdeI/BamHI* restriction sites. In JIP3, a single Lys to Arg difference occurs between mouse and human isoform in this N-terminal part. All fragments were produced in *Escherichia coli* Rosetta as N-terminus His-thioredoxin tag fusion proteins. Cells were collected after induction with 0.5 mM IPTG 5 h at 37 °C. Frozen bacteria were suspended in 50 mM Tris pH 8.0, 500 mM NaCl, 5% glycerol, 1 mM DTT, 25 mM imidazole pH 8.0, 0.5 mM EDTA and anti-proteases. After cell disruption by sonication, the lysate was ultracentrifuged at 40 000 rpm for 40 min at 4 °C and the supernatant was loaded on a His-Trap 5 mL column (GE Healthcare) equilibrated with 50 mM Tris pH 8.0, 500 mM NaCl, 5% glycerol, 1 mM DTT, 0.5 mM EDTA and 25 mM imidazole pH 8.0. Protein elution was performed in the same buffer containing 500 mM imidazole. Removal of the His-thioredoxin tag was performed by an overnight incubation with thrombin followed by a second Ni affinity. Finally, all cleaved fragments were purified by a gel filtration on a HiLoad 16/60 Superdex 75 column (GE Healthcare) in 20 mM Hepes pH 7.5 and 200 mM NaCl. All JIP3 and JIP4 fragments were stored at −80 °C. cDNAs encoding the JIP3-RH1-Lz3 fragment (residues 22–100) was cloned, expressed and purified similarly as the other JIP3/4 fragments.

The recombinant expression of human myosin 5a (myo5a) globular tail domain (GTD; residues 1463–1855) was performed in *Escherichia coli* Rosetta using a pProEx HTB vector containing an N-terminal 6x Histidine tag and a rTEV cleavage. Bacteria cells were collected after induction with 0.3 mM IPTG, O/N at 20 °C. Frozen bacteria were suspended in 50 mM Tris pH 8.0, 500 mM NaCl, 5% glycerol, 1 mM DTT, 25 mM imidazole pH 8.0, 2 mM MgCl_2_, 0.5 mM EDTA and anti-proteases. After cell disruption by sonication, the lysate was ultracentrifuged at 40 000 rpm for 40 min at 4 °C and the supernatant was loaded on a His-Trap 5 mL column (GE Healthcare) equilibrated with 50 mM Tris pH 8.0, 500 mM NaCl, 5% glycerol, 1 mM DTT, 0.5 mM EDTA, 2 mM MgCl_2_, and 25 mM imidazole pH 8.0. Protein elution was performed in the same buffer containing 500 mM imidazole. Then, myo5a-GTD was purified by a gel filtration on a HiLoad 16/60 Superdex 75 column (GE Healthcare) in 20 mM Hepes pH 8.0, 150 mM NaCl, 50 μM TCEP and 5% Glycerol, and stored at −80 °C.

### Biophysical characterization

Protein samples were analysed by Size-Exclusion Chromatography (SEC) coupled with Multi-Angle Laser Light Scattering (MALS). All JIP3/4 fragments were loaded on a Superdex 200 10/300 GL increase column (GE Healthcare) equilibrated with 20 mM Hepes pH 7.5 and 200 mM NaCl using a flow rate of 0.5 ml/min on a HPLC system (Shimadzu). Static light scattering and UV absorbance at 280 nm signals were measured with a MiniDAWN TREOS (Wyatt Technology) and UV detector SPD-M20A (Shimadzu), respectively. Data was analysed using ASTRA® 6.1 software (Wyatt Technology). The circular dichroism (CD) spectra of JIP3/4 fragments were recorded on a Jasco J-810 spectrometer (Japan Spectroscopic Co., Tokyo, Japan) at room temperature in 20 mM sodium phosphate pH 7.5 and 200 mM NaF for all proteins. All of the spectra were recorded using a 0.1-mm path length quartz cell. Each spectrum is a continuous scanning of 50 nm/min, in the range of 260–185 nm, averaged by 6 scans. Secondary structure analysis was performed using CONTINLL software^68^. Melting temperatures were determined by Differential Scanning Calorimetry (DSC) using the MicroCal PEAQ-DSC Automated (Malvern). JIP3/4 fragments (at 20 µM in 20 mM Hepes pH 7.5, 200 mM NaCl) were placed in a cell and heated from 25 °C to 85 °C using a 1 °C/min rate. The thermal behaviour of samples was recorded and analysed using MicroCal PEAQ-DSC software.

### Analytical ultracentrifugation

All sedimentation velocity experiments were performed on an analytical ultracentrifuge XLI (Beckman Coulter, Palo Alto, USA) with an An-50 Ti rotor at 20 °C. For the characterization of the self-association of the JIP3-Nter dimer, experiments were done with a fluorescence detection system (AVIV Biomedical, Lakewood, NJ). Two-channels 12 mm path-length Epon charcoal-filled centrepieces were used. JIP3 protein was labelled with Monolith NT.115 Protein Labelling Kit BLUE NHS (Nanotemper Technologies) following the manufactures’ instructions. Titration experiments were performed with the RH1-LZI and LZI fragments at concentrations ranging from 460 nM to 1.1 nM and from 770 nM to 2.4 nM, respectively. For protein sample from 1.1 nM to 60 nM, only labelled protein was used; contrary to higher concentrated samples that corresponded to the mixture of labelled protein (60 nM) with unlabelled protein to reach the total concentration. 400 μL of JIP3 samples were centrifuged at 50,000 rpm (181,827 g). Sedimentation profiles were collected every 4 min and analysed as described^69–71^.

For the JIP3:Myo5 formation study, experiments were performed with the absorbance detection system. JIP3-RH1-LZI and myo5a-GTD were firstly dialyzed in the interaction buffer (20 mM Hepes pH 7.5, 150 mM NaCl, 2 mM MgCl_2_ and 1 mM TCEP). JIP3-RH1-LZI (37 µM, red line), myo5a-GTD (12.5 μM; blue line) and two mixtures with the two fragments: one with an excess of JIP3 over myo5a (22.5 μM JIP3-RH1-LZI fragment + 7.5 μM myo5a-GTD; black curve) and one with an excess of myo5a overs JIP3 (7.5 μM JIP3-RH1-LZI fragment + 22.5 μM myo5a-GTD, green line) were centrifuged at 42000 rpm. The 280 nm absorbance profiles were recorded every 10 min. All data were assigned by the Sedfit software^72^.

### Homology modelling and structural analysis

We submitted the sequence of the N-terminal part (residues 1–187) of JIP3 to the web portal for protein modelling by threading using Phyre2^38^. With a confidence of 99.8%, Phyre2 identified the crystal structure of the RH1 domain of RILP-like protein 2 (RILPL2; PDB code 4KP3^37^;), as the highest scoring template for the RH1 domain and the beginning of the LZI of JIP3. Phyre2 gave same results when the JIP4 N-terminal sequence is submitted. Although Phyre2 modelled the last part of the extreme N-terminus (residues 18–21), the linker (residues 72–77) and the first part of the LZI coiled coil (residues 78–98), we only conserved the RH1 domain of JIP3 (residues 22–76). Then, we modelled the LZI of JIP3 (residues 78–180) as a straight parallel dimeric coiled coil using CCbuilder2.0^39^. Thirdly, using the graphical software COOT^73^, the N-terminal part of JIP3 was manually built by associating the RH1 model (23–71) to the LZI model (78–180) with the linker residues in between (^72^ENQEHE^77^) modelled as a helix. Finally, geometry minimization refinement of the overall model was carried out with PHENIX^74^. Structure and protein-protein interaction analysis, as well as figures were performed using PyMOL graphical program^75^.

### Small angle X-ray scattering (SAXS)

SAXS measurements were done at the SWING beamline of the SOLEIL synchrotron at a working energy of 12.0 keV (λ = 1.033 nm) and detector to sample distance was 1.5 m. The samples were subjected to a size exclusion purification online with the HPLC system (Supplementary Fig. S3) connected to a quartz capillary placed under vacuum cell using an Agilent Bio SEC. 3–300 column^76^. The samples were injected at 6 mg/ml with the elution buffer containing 20 mM Hepes pH 7.1, 300 mM NaCl, 0.5 mM TCEP. Data reduction, averaging of identical frames corresponding to the elution peak and buffer subtraction were performed with the SWING in-house software Foxtrot. The radius of gyration (Rg) and forward intensity at zero angle I(0) were derived by the Guinier approximation using the software PRIMUS^77^. The maximum dimension (Dmax) and the Rg were derived from the pair distribution function P(r), calculated with the software GNOM^78^. The molecular weight was calculated with SAXSMow2, extending the integration limit to a q_m_ of 0.5 Å^−1^ ^41^.

In order to have a full model corresponding to the fragment produced for SAXS data comparison, residues 181–187, as well as Gly-Ser-His residues remaining after the thrombin cleavage were added manually to the RH1-LZ1 tandem model. Crysol was used to fit the theoretical scattering of this model to the experimental data^79^. To refine the model against the SAXS data, we used the DADIMODO server that allows to kept rigid domains, while other regions can be defined as flexible^43,44^. The RH1 domain (residues 26–66) and the LZI coiled coil (residues 78–176) were kept as rigid bodies, while the N-terminus (residues Gly-Ser-His-[22–25]), the linker and few residues before (residues 67–77), and the C terminus (residues 177–187) were allowed to move. Five independent refinements were done. The web interface of EOM 2.1^45^ was used to generate 10,000 structures using the model of the RH1-LZ1 tandem of JIP3 as template. Only the flexible linker (residues 67–77) were considered as a flexible region. Five independent runs of 100 cycles were run to estimate the variability in the distribution of the *Rg* values.

### Microscale Thermophoresis

JIP3-Nter fragments and mutant were labeled using the Protein Labeling Kit Blue-NHS (NanoTemper Technologies). The labeling reaction was performed according to the manufacturer’s instructions in the supplied labeling buffer applying a concentration of 20 μM protein (molar dye:protein ratio ≈ 3:1) at room temperature for 30 min. The labeled proteins were stocked in MST buffer (50 mM TrisHCl pH 7.8, 150 mM NaCl, 10 mM MgCl_2_, 0.05% Tween20). For the binding assays, the labeled JIP3-Nter fragments were adjusted to 25 nM with the assay buffer (20 mM hepes pH 7.5, 150 mM NaCl, 2 mM MgCl_2_, 0.5 mM TCEP, 0.05% tween). Myo5a-GTD was dialyzed in the assay buffer and a series of 16 1:1 dilution was prepared. For the measurement, each myo5a-GTD dilution was mixed with one volume of labeled JIP3-Nter fragments and mutant. The samples were loaded into Monolith NT.115 Capillaries MO-K022 (NanoTemper Technologies). MST was measured using a Monolith NT.115 instrument (NanoTemper Technologies) at 25 °C. Instrument parameters were adjusted to 20% LED power and medium MST power. Data of three independent replicates was analysed using the signal from an MST-on time at 4–5 s (MO.Affinity Analysis software version 2.3, NanoTemper Technologies).

### Accession codes

SAXS data has been deposited into the SASBDB database (http://www.sasbdb.org/) with entry codes SASBDB: SASDGV4 (JIP3-RH1-LZI) and SASDGW4 (JIP3-LZI).

## Supplementary information

Supplementary Info
