# Supplementary material for: Structural characterization of the RH1-LZI tandem of JIP3/4 highlights RH1 domains as a cytoskeletal motor-binding motif
Source: Sci Rep. 2019 Nov 5;9:16036. doi: 10.1038/s41598-019-52537-3 (PMC6831827; doi:10.1038/s41598-019-52537-3)
Supplement: Supplementary file 1 — Supplementary Info [file 41598_2019_52537_MOESM1_ESM.docx]

**Structural characterization of the RH1-LZI tandem of JIP3/4**

**highlights RH1 domains as a cytoskeletal motor-binding motif**

**Fernando Vilela^1^, Christophe Velours^1^, Mélanie Chenon^1^, Magali Aumont-Nicaise^1^,**

**Valérie Campanacci ^1^, Aurélien Thureau^2^, Olena Pylypenko^3^, Jessica Andreani ^1^,**

**Paola Llinas^1*^ & Julie Ménétrey^1*^**

^1^ Institute for Integrative Biology of the Cell (I2BC), CEA, CNRS, Univ. Paris‐Sud, Université Paris‐Saclay, 91198, Gif‐sur‐Yvette cedex, France. ^2^ Synchrotron SOLEIL, l'Orme des Merisiers, F-91410 Saint Aubin, France. ^3^ Structural Motility Team, Institut Curie, PSL Research University, CNRS, UMR 144, Paris, France.

**Correspondence Authors**: paola.llinas@i2bc.paris-saclay.fr and julie.menetrey@i2bc.paris-saclay.fr

**SUPPLEMENTARY INFORMATION**

**Table S1. Results of the HHpred remote homology search using JIP3_MOUSE positions 1-98 as a query against the PDB, Pfam and human proteome databases.**

| **Hit identifier** | **Hit description** | **Source** | **HHsearch probability** | **Sequence identity** | **Nb. of aligned positions** |
| --- | --- | --- | --- | --- | --- |
| NP_001305781.1 | JIP3 isoform 3 | Homo sapiens | 99.88 | 100% | 98 |
| NP_113618.2 | RILP | Homo sapiens | 99.81 | 28% | 97 |
| PF09744.9 | Jnk-SapK_ap_N (JIP3/JIP4 N-ter region) | Pfam family | 99.79 | 69% | 70 |
| NP_003962.3 | JIP4 isoform 3 | Homo sapiens | 99.75 | 78% | 92 |
| 4KP3_D | RILPL2 | Mus musculus | 99.71 | 21% | 75 |
| NP_001306172.1 | RILPL1 isoform 2 | Homo sapiens | 99.63 | 34% | 77 |
| NP_659495.1 | RILPL2 | Homo sapiens | 99.38 | 26% | 73 |

Only hits with HHsearch probability > 95% are displayed. Hits were filtered for redundancy so that each protein only appears once (as a single isoform) in the table. JIP, C-Jun-amino-terminal kinase-interacting protein; RILP, Rab-interacting lysosomal protein; RILPL, RILP-like protein

**Table S2. Biophysical characterization of the N-terminal part of JIP4.**

| **Fragment** | | | | **Sec-MALS** | | |
| --- | --- | --- | --- | --- | --- | --- |
| **name** | **Limit** | **Mw***  (kDa) | | **Mw**  (kDa) | **Rh**  (nm) | **f/f0** |
| **RH1-LZI** | [17-182] | | 20.2 | 57.8 ± 0.3 | 4.8 ± 0.3 | N.D. |
| **LZI** | [67-182] | | 14.7 | 41.2 ± 0.4 | 4.4 ± 0.3 | N.D. |
| **RH1** | [17-69] | | 6.3 | N.D. | | |

N.D. Not Determined; Rand., Random; * Mw is calculated considering the remaining sequence of the protease site after cleavage.

**Table S3. SAXS data collection and scattering-derived structural parameters of the RH1-LZI and LZI fragments of JIP3**

| **Data collection** | **RH1-LZI fragment** | **LZI fragment** |
| --- | --- | --- |
| Beamline | SWING | |
| Detector | EIGER-4M | |
| Wavelength (Å) | 1.0332 | |
| q range (Å) | 0.0048 to 0.8167 | |
| Sample-detector distance (m) | 1.5 | |
| Exposure time (ms) | 990 | |
| **Structural parameters** |  |  |
| **Gunier analysis** |  |  |
| I(0) (cm^-1^) | 0.055 ± 0.00017 | 0.038 ± 0.00013 |
| Rg (Å) | 63.19 ± 2.87 | 51.02 ± 0.32 |
| qRg limits | 0.54 -1.11 | 0.53-1.12 |
| **P(r) analysis** |  |  |
| I(0) (cm^-1^) | 0.0551 ± 0.00018 | 0.038 ± 0.00013 |
| Rg from P(r) (Å) | 66.14 ± 0.41 | 53.93 ± 0.36 |
| Dmax (Å) | 231 | 198 |
| q range | 0.0085-0.76 | 0.0085-0.7301 |
| **Molecular mass determination (kDa)** |  |  |
| Calculated from sequence | 20.3 | 14.8 |
| MM SAXSMoW^a^ | 42.3 | 33.4 |

^a^ Integration limit to q_m_=0.5

**Figure S1. Bioinformatics analysis of the N-terminal part of JIP3 and JIP4 homologs. (a)** Phylogenetic tree of the N-terminal part of the JIP3 and JIP4 homologs. The tree reconstruction is derived from a multiple sequence alignment of homologs of JIP3 and JIP4 retrieved from different metazoan species. JIP3-like sequences in invertebrates and JIP3 and JIP4 orthologs in vertebrates are indicated in purple, blue and red, respectively. The purple star indicates the probable position of the gene duplication leading to JIP3 and JIP4 homologs. The tree leaves are labelled with the NCBI gene index (gi) of the protein sequence followed by an abbreviated name of the corresponding species. For visualization purposes, the tree was rooted at the separation between vertebrates and invertebrates (including nematodes and insects). Four levels of thickness of tree branches indicate (from thickest to thinnest): internal branches with statistical support ≥ 90%; internal branches with statistical support ≥ 80 % but < 90%; internal branches with statistical support < 80%; external branches. Branch support was computed with PhyML^1^ using the non-parametric approximate likelihood ratio test (“SH-aLRT”)^2^. The scale bar for branch length shown in the top left corner represents the average number of substitutions per site. The tree was visualized with Dendroscope 3 using a phylogram representation^3^. **(b)** Multiple sequence alignment of JIP3 and JIP4 homologs for the region containing the RH1 and the LZI. The alignment was visualized with Jalview^4^. The alignment of the full-length sequences (of which only the N-terminal part is shown here) was used to build the phylogenetic tree in Fig S1.

**Figure S2. Secondary structure predictions for the N-terminal part of JIP3 and JIP4.** Predictions were performed on the GeneSilico Metaserver (<https://genesilico.pl/meta2>) which used various methods for protein secondary structure prediction. H letter (in red) indicates helix, while E letter (in green) indicates strands.

**Figure S3. Biophysical characterization of the JIP4 N-terminal fragments.** **(a)** Schematic representation of the different fragments of the N-terminal part of JIP4 used in this study. **(b)** Tricine-SDS-PAGE 14% showing the N-terminal fragments of both JIP3 and JIP4 used in this study. The full gel is shown and no contrast modification was done. **(c)** Superposition of the MALS curves of JIP4 N-terminal fragments.

**Figure S4.** **SAXS studies of the RH1-LZI (left) and LZI (right) fragments of JIP3**. **(a)** SEC-SAXS profiles. Plot showing I(0) (black) and Rg (orange) as a function of the frames collected during SEC-SAXS run. **(b)** Experimental SAXS profile. Inset, the Guinier fit in the qRg range of 0.53-1.12. **(c)** The dimensionless Krakty plot. **(d)** The distance distribution function P(r). **(e)** Comparison of the experimental SAXS profile (black) and the theoretical profile calculated by CRYSOL from the RH1-LZI model (pink) and the LZI model (yellow).

**Figure S5. Biophysical characterization of the RH1-lz3 fragment of JIP3.** **(a)** Schematic representation of the different fragments of the RH1-lz3 fragment of JIP3 used in MST experiments. **(b)** MALS curves for the JIP3-RH1-lz3 fragment (in orange) compared to those of JIP3-RH1-LZI (in black) and JIP3-LZI (in green).

**References**

1. Guindon, S. *et al.* New Algorithms and Methods to Estimate Maximum-Likelihood Phylogenies: Assessing the Performance of PhyML 3.0. *Systematic Biol* **59,** 307–321 (2010).

2. Anisimova, M., Gil, M., Dufayard, J.-F., Dessimoz, C. & Gascuel, O. Survey of Branch Support Methods Demonstrates Accuracy, Power, and Robustness of Fast Likelihood-based Approximation Schemes. *Systematic Biol* **60,** 685–699 (2011).

3. Huson, D. H. & Scornavacca, C. Dendroscope 3: An Interactive Tool for Rooted Phylogenetic Trees and Networks. *Systematic Biol* **61,** 1061–1067 (2012).

4. Waterhouse, A. M., Procter, J. B., Martin, D. M., Clamp, M. & Barton, G. J. Jalview Version 2—a multiple sequence alignment editor and analysis workbench. *Bioinformatics* **25,** 1189–1191 (2009).
